# Proteomic Analysis of Lymphoblastoid Cells Derived from Monozygotic Twins Discordant for Bipolar Disorder: A Preliminary Study

**DOI:** 10.1371/journal.pone.0053855

**Published:** 2013-02-07

**Authors:** An-a Kazuno, Kenji Ohtawa, Kaori Otsuki, Masaya Usui, Hiroko Sugawara, Yuji Okazaki, Tadafumi Kato

**Affiliations:** 1 Laboratory for Molecular Dynamics of Mental Disorders, Brain Science Institute, RIKEN, Saitama, Japan; 2 Research Resources Center, Brain Science Institute, RIKEN, Saitama, Japan; 3 Department of Psychiatry, Tokyo Metropolitan Matsuzawa Hospital, Tokyo, Japan; Chiba University Center for Forensic Mental Health, Japan

## Abstract

Bipolar disorder is a severe mental illness characterized by recurrent manic and depressive episodes. In bipolar disorder, family and twin studies suggest contributions from genetic and environmental factors; however, the detailed molecular pathogenesis is yet unknown. Thus, identification of biomarkers may contribute to the clinical diagnosis of bipolar disorder. Monozygotic twins discordant for bipolar disorder are relatively rare but have been reported. Here we performed a comparative proteomic analysis of whole cell lysate derived from lymphoblastoid cells of monozygotic twins discordant for bipolar disorder by using two-dimensional differential in-gel electrophoresis (2D-DIGE). We found approximately 200 protein spots to be significantly differentially expressed between the patient and the co-twin (*t* test, *p*<0.05). Some of the proteins were subsequently identified by liquid chromatography tandem mass spectrometry and included proteins involved in cell death and glycolysis. To examine whether these proteins could serve as biomarkers of bipolar disorder, we performed Western blot analysis using case–control samples. Expression of phosphoglycerate mutase 1 (PGAM1), which is involved in glycolysis, was significantly up-regulated in patients with bipolar disorder (*t* test, *p*<0.05). Although PGAM1 cannot be regarded as a qualified biomarker of bipolar disorder from this preliminary finding, it could be one of the candidates for further study to identify biomarkers of bipolar disorder.

## Introduction

Bipolar disorder is a severe mental illness characterized by recurrent manic and depressive episodes. It affects approximately 1% of the general population. In bipolar disorder, contributions of genetic and environmental factors have been indicated by family and twin studies [1]. Patients with this disease have a high relapse rate, and lifelong treatment with mood stabilizers such as lithium is often needed [2]. Past studies of bipolar disorder have focused on monoamines and intracellular signal transduction pathways as related to the effects of psychotropic drugs [3], [4], but recent studies focus more on neuroplasticity or resilience based on reports of neuroprotective effects of mood stabilizers [5]. Recent genome-wide association studies identified new candidate genes, such as *CACNA1C* [6], [7], but the effects are relatively small.

Bipolar disorder is often misdiagnosed as depression or schizophrenia, and delayed diagnosis and treatment worsen the course of illness [8]. Thus, early diagnosis is important to prevent deterioration; however, no biomarkers for bipolar disorder are available yet. Identification of biomarkers would be indispensable for early diagnosis.

Much research on biomarkers in psychiatric diseases has been published [9], [10]. Though several candidate biomarkers were identified from postmortem brains, these studies are undermined by many confounding factors, including cause of death, postmortem interval, and brain pH [11]. Postmortem interval affects degradation of mRNAs and protein levels [12], [13], [14], [15] and significantly influences phosphorylation of signaling proteins [16]. In particular, the influence of medication cannot be ignored because a number of proteins are affected by mood stabilizers, antipsychotics, or antidepressant medication [17], [18], [19]. Additionally, large interindividual variations hamper the identification of biomarkers.

Biomarker research in other tissues such as serum, plasma, cerebrospinal fluids, saliva, and urine have also been performed [20], [21] but has not led to a diagnostic test.

The concordance rate of bipolar disorder between monozygotic twins is approximately 70% [22], [23]. By comparing monozygotic twins discordant for bipolar disorder, biochemical differences associated with bipolar disorder might be detected without interference from interindividual genetic variation [24].

Although several genome, transcriptome, and epigenome analyses in monozygotic twins discordant for bipolar disorder and other diseases have been reported [24]–[29], proteomic analysis has not been applied to identifying the difference between monozygotic twins discordant for bipolar disorder, possibly due to technical difficulties. Because transcript levels do not completely correlate with protein expression levels [30], [31] and aberrant post-translational modifications can cause disease, proteomic analysis is needed to supplement transcriptome and epigenome analyses.

In the present study, we performed proteomic analysis of lymphoblastoid cells derived from monozygotic twins discordant for bipolar disorder.

## Materials and Methods

### Subjects

For 2D-DIGE, we used lymphoblastoid cells derived from a pair of 42-year-old male monozygotic twins discordant for bipolar disorder. We initially examined their monozygosity by genotyping microsatellite repeat markers [25] and later confirmed it by single nucleotide polymorphism array [29].

For a case–control study of differentially expressed proteins using Western blot analyses, we used lymphoblastoid cells derived from eight unrelated patients with bipolar I disorder (BPI) (four men and four women, 35.6±9.0 years old [mean ± SD], Japanese) and eight unrelated control subjects (six men and two women, 36.9±10.0 years old [mean ± SD], Japanese). Table 1 gives detailed information for each subject used in Western blot analysis. Patients and controls with a history of alcohol or illicit drug abuse were excluded from the study. The patients were treated with various medications. Diagnoses were made by the consensus of two senior psychiatrists using the Diagnostic and Statistical Manual of Mental Disorders, Fourth Edition (DSM-IV) criteria. Controls were selected from among students, nurses, office workers, and doctors in participating institutes, their friends, and other volunteers. A senior psychiatrist interviewed control subjects and found that they did not have major mental disorders. Written informed consent was obtained from all subjects. The ethics committees of RIKEN approved the study.

**Table 1:** Characteristics of the subjects.

ID	Age	Sex	Diagnosis	Age at onset	Family history of bipolar disorder within first degree relatives	Medication
B1	40	F	Bipolar I disorder with psychotic features	24	Yes	Li, VPA, CBZ, HP, CP
B2	23	F	Bipolar I disorder	23	No	Li, VPA, PAR
B3	46	F	Bipolar I disorder	27	Yes	VPA, THY
B4	26	M	Bipolar I disorder, rapid cycling	24	Yes	Li
B5	38	M	Bipolar I disorder	35	No	VPA, OLA
B6	40	M	Bipolar I disorder	25	No (three had major depression)	Li, VPA
B7	27	F	Bipolar I disorder with psychotic features	24	No	Li, VPA
B8	45	M	Bipolar I disorder	20	No	Li
C1	36	M	-		No	-
C2	29	M	-		No	-
C3	34	M	-		No	-
C4	29	M	-		No	-
C5	27	F	-		No	-
C6	52	M	-		No	-
C7	52	M	-		No	-
C8	33	F	-		No	-

Li: lithium carbonate, VPA, sodium valproate, CBZ, carbamazepine, HP, haloperidol, CP, chlorpromazine, PAR, paroxetine, THY, levothyroxine, OLA, olanzapine.

### Cell culture and extraction of proteins

Lymphocytes were separated from the peripheral blood and transformed by Epstein-Barr virus using previously described standard techniques [32]. These cells were cultured and kept frozen until experiments. Lymphoblastoid cells were cultured in RPMI 1640 medium (Sigma-Aldrich, St. Louis, MO, USA) containing 10% fetal bovine serum (Sigma-Aldrich), 50 U/mL penicillin, 50 μg/mL streptomycin (GIBCO, Invitrogen/Life Technologies Corporation, Grand Island, NY), and 60 μg/mL tylosin solution (Sigma-Aldrich). Cells were cultured at approximately 1×10^8^ cells. Total proteins were extracted from the lymphoblastoid cells using the Q-proteome mammalian protein preparation kit (Qiagen, QIAGEN, Hilden, Germany). After the supernatant was precipitated with acetone, the pellet was dissolved using the lysis buffer (7 M urea, 2 M thiourea, 4% CHAPS, 10 mM Tris-HCl [pH 8.5]) (GE Healthcare Bio-Sciences, San Francisco, CA, USA).

### Two-dimensional difference gel electrophoresis (2D-DIGE) and imaging analyses

Protein concentration was determined by the Bradford method using a protein assay kit (Bio-Rad, Hercules, CA, USA) and the Pierce 660 nm protein assay (Pierce, Rockford, IL, USA). Proteins were set to a final concentration of 5 mg/mL with the lysis buffer and labeled separately with 400 pmol of CyDye (Cy3 or Cy5) (GE Healthcare Bio-Sciences), vortexed, and incubated on ice in the dark for 30 min. A mixed sample composed of equal amount of proteins from the patient and the co-twin was labeled with Cy2 and used as an internal standard. After 30 min, the labeling reaction was stopped with 10 mM lysine. To avoid the possible effect of labeling efficiency, the dyes were swapped for each experiment using three gels. Labeled proteins were subjected to SDS-PAGE analysis, and the gels were scanned with the Typhoon 9400 scanner (GE Healthcare Bio-Sciences) at the wavelengths corresponding to each CyDye, namely 480 nm (Cy2), 532 nm (Cy3), and 633 nm (Cy5). A 50 μg portion of each Cy3-, Cy5-, and Cy2-labeled sample was combined. Nonlinear IPG strips (pH 3–10, 18 cm long; GE Healthcare Bio-Sciences) were rehydrated for 12 h at 50 mA per strip with the sample solution on an IPGphor isoelectric focusing unit (GE Healthcare Bio-Sciences). Three mixed samples were separated by isoelectric focusing on an IPGphor isoelectric focusing unit at 0.5 V⋅h, 0.8 V⋅h, 13.5 kV⋅h, and 21 kV⋅h at 20°C and a maximum current setting of 50 μA per strip. The second dimension was run on 12.5% acrylamide gels in a SE600 (GE Healthcare Bio-Sciences) at 45 mA per gel. To avoid artifacts, three gels of same condition were simultaneously run for each experiment. Gels were scanned directly between low-fluorescence glass plates with the Typhoon 9400 (GE Healthcare Bio-Sciences) scanner at the three wavelengths specific for the CyDyes. The resolution was approximately 100 μm. Determination of protein spot abundance was performed using the DeCyder 2D Ver. 6.0 software (GE Healthcare Bio-Sciences). Spots were automatically detected. Spot editing (separation of two spots) or deleting (artifacts) was performed manually. The three CyDye–labeled forms of each spot were co-detected within each gel. Ratios between sample and internal standard abundances were calculated for each protein spot with the Differential In-gel Analysis (DIA) module. Inter-gel variability was corrected by matching and normalization of the internal standard spot maps by the Biological Variance Analysis (BVA) module of the DeCyder software and incorrectly matched spots were manually eliminated or corrected if possible. During the spot detection, the estimated number of spots was set at 4000. Protein spots that showed a statistically significant intensity in Student's *t* test were accepted as being differentially expressed between the extracts under comparison among these. Protein spots showing at least 1.25-fold changes (*p*<0.05) in intensity were selected for next steps.

### Protein identification by mass spectrometry and database search

The preparative gels were stained with a SYPRO® ruby (Invitrogen/Life Technologies Corporation) and scanned with the Typhoon 9400. Protein spots that showed differences in relative fluorescence were excised from the gel using the automated spot picker (GE Healthcare Bio-Sciences). The picked gel pieces were destained with 50% CH_3_CN in a 50 mM NH_4_HCO_3_ solution. After removal of the supernatant, cysteine residues were reduced with dithiothreitol and carbamidomethylated with iodoacetamide. In-gel trypsin digestion was performed at 37°C overnight, using sequencing grade modified trypsin (Promega, Southampton, UK) reconstituted in 100 mM NH_4_HCO_3_. The trypsinized gel was rinsed three times in extraction buffer (5% trifluoroacetic acid in 50% CH_3_CN and 50% H_2_O). The trypsinized peptides solution was dried by speed vacuum, suspended in 2% CH_3_CN with 0.1% trifluoroacetic acid, and analyzed by LTQ (Fisher Scientific, Waltham, MA) liquid chromatography/linear ion trap mass spectrometry (LC-MS/MS) system. Their corresponding proteins were searched using the program Mascot database-searching software (Matrix Science, London, UK), which accesses protein identification by matching mass spectroscopy data with the protein databases NCBI (http://www.ncbi.nlm.nih.gov) and UniProt (http://www.uniprot.org/uniprot). Identification criteria included a Mascot score >45 (selected based on a corrected *p*-value <0.05).

### Functional grouping of altered proteins

The IPA software (Ingenuity Systems, Redwood City, CA) was used to identify the key biological relationships and functions of differentially expressed proteins and their interaction networks. For pathway analysis, Swiss-Prot and GenBank accession numbers were used to catalogue the identified protein into known interaction pathways based upon the Ingenuity Pathway Knowledge Base (IPKB). IPA classification data were derived from the published literature in a systematic way using a comprehensive ontology of functional annotations and protein–protein interaction data. The most significant interaction networks, biological functions and pathway associated with the differentially expressed proteins were identified. To confirm biological functions and gene ontology annotation between identified proteins, we used bioinformatics resources, PANTHER (http://www.pantherdb.org/) and DAVID (http://david.abcc.ncifcrf.gov/). DAVID program uses a modified Fisher's exact *p* value (EASE score) to rank gene clusters by statistical overrepresentation of individual genes, based on the co-occurrence/enrichment of the category within the gene list relative to all genes in the same category on the study.

### Western blot analyses for validation

Lymphoblastoid cells in a case–control study were individually cultured. Total proteins were individually extracted using Q-proteome Mammalian Protein prep kit (Qiagen), and protein concentrations were determined by the methods mentioned above. Equal concentration (5 μg per lane) of proteins from control and case samples were separated by 12% or 4–15% SDS-polyacrylamide gel electrophoresis and transferred onto Immun-Blot PVDF membranes (Bio-Rad) using a mini Trans-blot Cell (Bio-Rad). After transfer, the blotted membrane were blocked with 4% w/v ECL Advance Blocking Reagent (GE Healthcare Bio-Sciences) in phosphate-buffered saline containing 0.1% Tween20 (PBST) (MP Biomedicals Inc., Santa Ana, CA) at 4°C overnight and incubated with primary antibody in PBST with 4% w/v skim milk for 1 h at room temperature. PSME1, WARS, and PGAM1 were chosen for quantification by Western blot analysis. As an internal control, NM23A was used because there was no significant difference of protein levels of NM23A between patient and the co-twin in 2D-DIGE. Primary antibodies were as follows: rabbit antibody against human PSME1 (Calbiochem, La Jolla, CA), mouse antibody against human WARS (Abnova, Taipei City, Taiwan), goat antibody against human PGAM1 (Novus Biologicals, Littleton, CO), and rabbit monoclonal and polyclonal against human NM23A (Abcam, Cambridge, MA). The blotted membranes were washed in TBST and incubated at room temperature with each secondary antibodies, Alexa Fluor 488–labeled donkey anti-goat IgG antibody (Molecular Probes/Life Technologies Corporation), Cy5-conjugated affiniPure donkey anti rabbit IgG antibody and Cy3-conjugated affiniPure donkey anti mouse IgG antibody (Jackson Immuno Research, West Grove, PA). The membranes were directly scanned with the Molecular Imager FX (Bio-Rad). Protein bands were analyzed to give a quantitative estimation of intensity change using the Quantity One Software (Bio-Rad) adapted to the Molecular Imager FX. To estimate the relative molecular weight of each protein, molecular markers, Dual Color Precision Plus Protein Standards (Bio-Rad) and ECL Plex Fluorescent Rainbow Markers (GE Healthcare Bio-Sciences) were used. Preliminary experiments indicated that amounts of these proteins in the lysates of lymphoblastoid cells were within the linear range of detection.

## Results

### Detection of spots differentially expressed between monozygotic twins discordant for bipolar disorder by 2D-DIGE

First, we extracted total protein from cultured lymphoblastoid cells derived from a pair of monozygotic twins discordant for bipolar disorder. The total protein for each twin was separately labeled with different CyDyes (Cy3 or Cy5), and dyes were swapped between gels. A mixed sample composed of an equal amount of proteins from the patient and the co-twin was labeled with Cy2 and used as an internal standard. These processes minimized gel-to-gel variation and improved protein spot statistics at the analysis stage. These labeled proteins were mixed and analyzed by 2D-DIGE. To detect robust differences between the patient and the co-twin, we performed 2D-DIGE. To avoid artifacts, four gels of same condition were simultaneously run for each experiment. Three gels were used as analytical gels to detect differentially expressed spots between the patient and the co-twin, and the remaining gel was used as preparative gel for picking out differentially expressed spots. We performed 2D-DIGE and liquid chromatography tandem mass spectrometry (LC-MS/MS) analyses in quadruplicate experiments using protein samples independently extracted from different aliquots of cell culture.

Using 2D-DIGE and DeCyder Ver.6.0 image analysis software, approximately 3200 protein spots were separated (3220±51 [mean ± SD]). The observed spot pattern images in each gel were very similar among quadruplicate experiments. The protein spots were selected if the intensity difference between the affected and nonaffected twin was larger than 1.25-fold (absolute value >1.25 or absolute value <−1.25). Approximately 200 spots (211±115, quadruplicate) were found to be significantly differentially expressed (*p*<0.05, Student's *t* test) between the twins per experiment.

### Identification of spots by LC-MS/MS

The proteins in preparative gels were stained by SYPRO Ruby after electrophoresis, and the preparative gels were performed matching to analytical gels. The largest 100 spots out of the 200 differentially expressed protein spots were picked from each preparative gel and were considered suitable for subsequent analysis by LC-MS/MS. The spots were chosen sequentially from those with a large absolute value fold change. The 68 spots, averaged for quadruplicate values, were successfully identified as unique proteins through LC-MS/MS and are listed in Table 2. Since Epstein-Barr virus–transformed lymphoblastoid cells were used in this study, all immunoglobulin and B-cell–related proteins were removed from the analysis. Moreover, we also removed keratin and trypsin-related proteins because of the possibility of experimental contamination. Table 2 shows only proteins identified by LC-MS/MS in common with each experiment. Proteins identified twice or more with Mascot search results (ion scores of higher than 45) included PSME1, RPLP0, TPI1, ALDOC, ANXA4, PGAM1, and WARS. Fifty-three proteins had high ion scores and were identified at least twice in four experiments.

**Table 2:** Proteins differentially expressed in monozygotic twin discordant for BP identifed by LC-MS/MS.

			Exp.1		Exp.2		Exp.3		Exp.4	
			Fold change		Fold change		Fold change		Fold change	
Gene names	Acc. No.	Protein names	(BP/CT)	*p* – value	(BP/CT)	*p* – value	(BP/CT)	*p* – value	(BP/CT)	*p* – value
PSME1	Q06323	Proteasome activator complex subunit 1	−1.42	0.00013	−1.44	0.00018	−1.3	0.0079	−1.91	0.0056
RPLP0	P05388	60S acidic ribosomal protein P0	−1.34	0.00031	−1.39	0.03	−1.45	0.0053	−1.31	0.0059
TPI1	P00939	Triosephosphate isomerase	1.95	0.034	3.17	7.10E-05	1.68	0.015	1.3	0.015
ALDOC	P09972	Fructose-bisphosphate aldolase C	−1.47	0.0024	−1.35	0.0013	−1.26	0.0059		
ANXA4	P09525	Annexin A4	−1.96	0.00022	−1.62	0.00011	−1.35	0.00029		
PGAM1	P18669	Phosphoglycerate mutase 1	1.9	0.0023	1.59	0.0001			2.12	0.0035
WARS	P23381	Tryptophanyl-tRNA synthetase, cytoplasmic	1.6	0.00037	1.55	0.00023			1.28	0.00062
ACADS	P16219	Short-chain specific acyl-CoA dehydrogenase, mitochondrial					−1.56	0.00014	−1.37	0.0006
ALDH2	P05091	Aldehyde dehydrogenase, mitochondrial					−1.26	0.018	−1.29	0.01
ALDOA	P04075	Fructose-bisphosphate aldolase A			1.48	0.0023			1.29	0.0055
ANXA5	P08758	Annexin A5					−1.25	0.0046	−1.29	0.007
APOA1BP	Q8NCW5	Apolipoprotein A-I-binding protein	2.56	1.90E-06	2.16	0.00023				
ATP5A1	P25705	ATP synthase subunit alpha, mitochondrial	−1.61	0.00048			−1.33	6.70E-06		
C19orf10	Q969H8	UPF0556 protein C19orf10	1.66	0.001	2.16	0.0012				
CACYBP	Q9HB71	Calcyclin-binding protein			−1.99	0.00075	−11.74	0.00023		
CAPZB	P47756	F-actin-capping protein subunit beta	−1.56	0.00021	−1.36	7.10E-06				
CASP3	P42574	Caspase-3	1.49	0.00011	1.44	0.0054				
CMPK1	P30085	UMP-CMP kinase	2.9	1.40E-05	3.22	0.00029				
CORO1A	P31146	Coronin-1A	1.31	0.0013	1.36	0.0029				
DNAJB11	Q9UBS4	DnaJ homolog subfamily B member 11	1.37	0.00032	1.41	0.0052				
ECH1	Q13011	Delta(3,5)-Delta(2,4)-dienoyl-CoA isomerase, mitochondrial	1.91	0.0014	1.74	0.00016				
ENO1	P06733	Alpha-enolase	−1.62	0.049	−1.58	0.012				
ETFB	P38117	Electron transfer flavoprotein subunit beta	1.27	0.019	1.36	0.00099				
GALE	Q14376	UDP-glucose 4-epimerase	−1.35	0.0051			−1.33	0.044		
GAPDH	P04406	Glyceraldehyde-3-phosphate dehydrogenase	−1.38	0.0038			−1.33	0.044		
HIST2H4B	P62805	Histone H4	2.07	0.0038	1.95	0.0014				
HNRNPM	P52272	Heterogeneous nuclear ribonucleoprotein M					1.46	0.0033	1.4	0.0023
HSPA5	P11021	78 kDa glucose-regulated protein	3.18	0.00012	3.02	5.50E-06				
HSPB1	P04792	Heat shock protein beta-1	1.58	0.0001	1.46	0.0071				
LDHA	P00338	L-lactate dehydrogenase A chain			1.26	0.035			5.6	0.0042
LGALS3	P17931	Galectin-3	−1.78	0.00033	−1.99	0.00075				
NANS	Q9NR45	Sialic acid synthase	1.46	0.0012	1.62	1.60E-05				
NDUFS3	O75489	NADH dehydrogenase [ubiquinone] iron-sulfur protein 3, mitochondrial	1.58	0.0001	1.46	0.0071				
NPM1	P06748	Nucleophosmin	−1.53	0.00074	−1.42	0.0042				
OTUB1	Q96FW1	Ubiquitin thioesterase OTUB1	1.52	6.00E-05	1.47	0.0043				
P4HB	P07237	Protein disulfide-isomerase	1.47	0.00017	1.69	0.0029				
PACAP	Q8WU39	Plasma cell-induced resident endoplasmic reticulum protein	2	0.011	2.01	0.0077				
PCBP1	Q15365	Poly(rC)-binding protein 1	−1.47	0.0024	−1.35	0.0013				
PDIA3	P30101	Protein disulfide-isomerase A3	1.98	8.60E-06	2.09	0.0017				
PDIA6	Q15084	Protein disulfide-isomerase A6	1.58	0.00042	1.75	1.20E-05				
PHGDH	O43175	D-3-phosphoglycerate dehydrogenase	2.05	0.003	3.13	0.0001				
PITHD1	Q9GZP4	PITH domain-containing protein 1	2.15	0.012	2.13	0.011				
PKM2	P14618	Pyruvate kinase isozymes M1/M2			1.36	0.0029			1.36	0.045
PNPO	Q9NVS9	Pyridoxine-5′-phosphate oxidase	−1.74	0.0085	−1.41	0.001				
POLR2E	P19388	DNA-directed RNA polymerases I, II, and III subunit RPABC1	1.65	0.00025	2.12	0.00013				
PRDX2	P32119	Peroxiredoxin-2	2.9	1.40E-05	3.22	0.00029				
PSMB1	P20618	Proteasome subunit beta type-1	−1.77	0.0093			−1.29	0.00032		
SARS	P49591	Seryl-tRNA synthetase, cytoplasmic	1.31	0.0013	1.36	0.0029				
SERPINB1	P30740	Leukocyte elastase inhibitor	1.37	0.00032	1.41	0.0052				
SSR4	P51571	Translocon-associated protein subunit delta	1.67	0.0059	1.77	0.00018				
STMN1	P16949	Stathmin	−2.02	6.90E-05	−1.53	3.70E-05				
UCHL1	P09936	Ubiquitin carboxyl-terminal hydrolase isozyme L1	2.5	1.90E-05	2.16	0.00023				
VDAC1	P21796	Voltage-dependent anion-selective channel protein 1	1.43	0.0013	2.29	0.00023				

### Functional grouping of altered proteins

**Figure 1:**
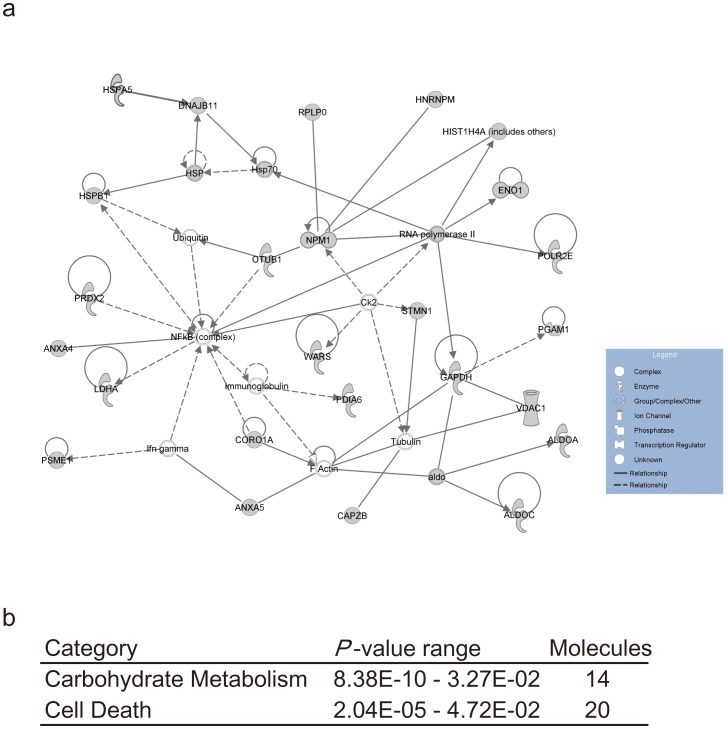
Gene networks showing interrelationships between differentially expressed genes in the twin with bipolar disorder.

To explore the biological function (protein-interaction network) of the 53 differentially expressed proteins, we performed protein classification using the Ingenuity Pathway Knowledge Base software. A data set containing the gene symbols by The HUGO Gene Nomenclature Committee was uploaded into the application. Each protein identifier was converted to its gene identification and mapped to its corresponding gene object in the Ingenuity Pathway Knowledge Base. These genes were overlaid onto a global molecular network developed from information contained in the Ingenuity pathways analysis (IPA) knowledge base, which is based entirely on findings reported in the literature. Networks of these focus genes were algorithmically generated based on their connectivity. One of the 53 proteins was omitted because it was not included in the database, and the remaining 52 proteins were mapped onto mainly three networks. The largest network, having the highest score, is associated with carbohydrate metabolism, neurological disease, and skeletal and muscular disorders (Fig. 1A). This network includes ALDOA, ALDOC, ANXA4, ANXA5, CAPZB, CORO1A, DNAJB11, ENO1, GAPDH, HIST1H4A (includes others), HNRNPM, HSPB1, LDHA, NPM1, OTUB1, PDIA6, PGAM1, POLR2E, PRDX2, PSME1, RNA polymerase II, RPLP0, STMN1, VDAC1, and WARS. Next, the 53 proteins were classified according to biological function and canonical pathway. The categories pertained to carbohydrate metabolism (8.38E-10–3.27E-02, 14 molecules) and cell death (2.04E-05–4.72E-02, 20 molecules) (Fig. 1B). Pathway analysis and gene ontology classification using PANTHER and DAVID were conducted on the same protein IDs. These analyses also showed pathways and categories associated with glycolysis and anti-apoptosis. Taken together, most proteins identified in the present study were related to glycolysis and neurological diseases.

### Case–control study by Western blot analysis

**Figure 2:**
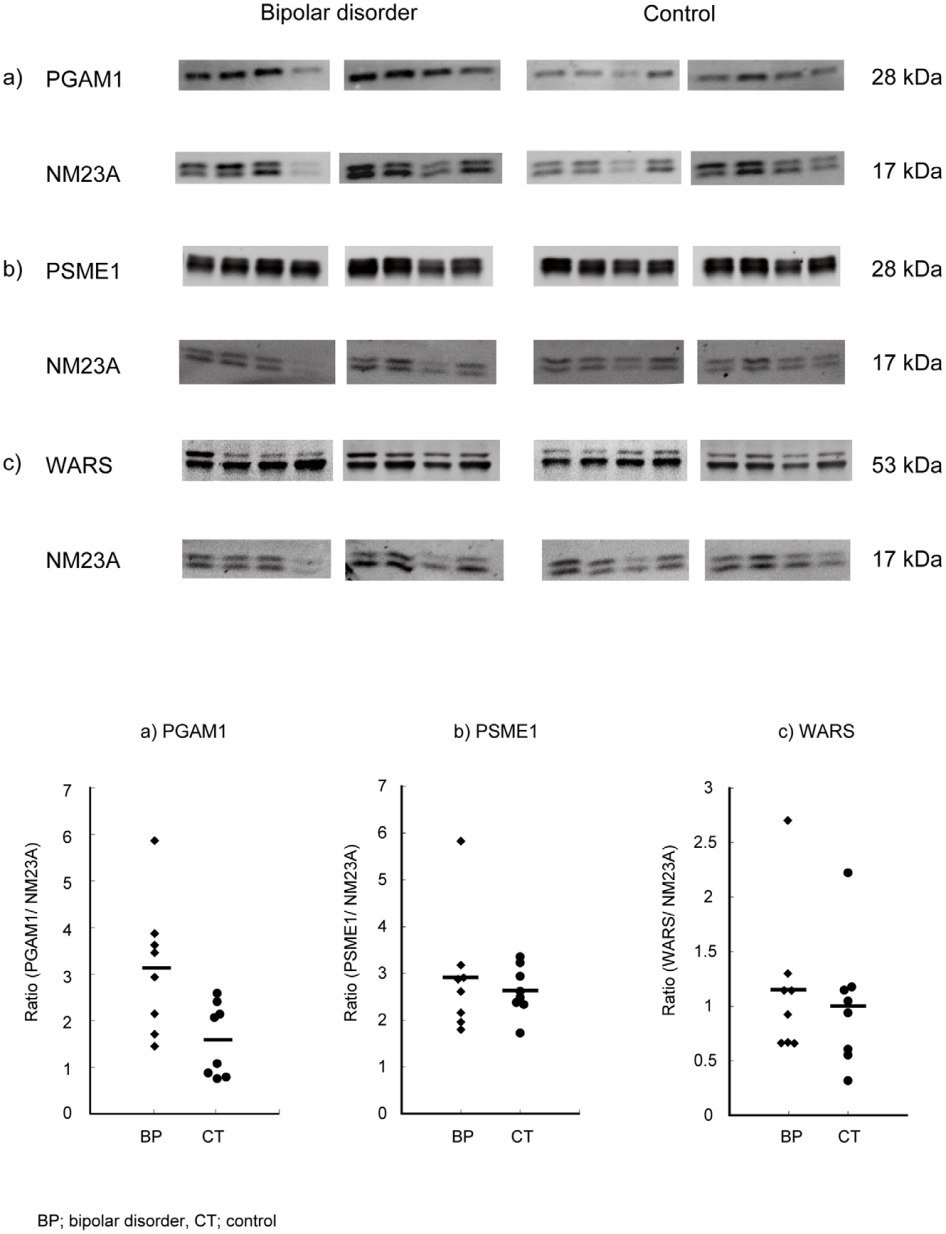
Protein expression validation by Western blot analysis with an internal standard, NM23A. Proteins from each of the eight pairs of samples were separated by SDS-PAGE and transferred to PVDF membranes. Proteins were immunodetected using the respective primary antibodies and fluorescent secondary antibodies. Signals were captured with FX and signal intensity and shown by the. a) PGAM1, b) PSME1, c) WARS. Scatter plots show the ratio of each protein to an internal standard protein, NM23A, measured by densitometric scanning of the band intensities. The *p* values were calculated using a *t* test in all proteins. Number of the subjects is 8 for bipolar disorder and 8 for controls, respectively. The absolute band intensity for the PGAM1 was also significantly higher in patients with bipolar disorder (0.93+/−0.23 [mean +/− standard deviation] [arbitrary unit]) than control subjects (0.39+/−0.18, p<0.0005).

We postulated that the differentially expressed proteins might be candidate biomarkers for bipolar disorder. To validate the findings from the proteomic profiling study and to examine the possibility of biomarkers for bipolar disorder, Western blot analyses were performed using a case–control sample set consisting of eight subjects with bipolar disorder and eight healthy control subjects. To compare the protein levels across individuals, protein concentration was measured by the Bradford method, and equal amounts of proteins were loaded onto the gels. Commercially available antibodies for the candidate 7 proteins (PSME1, RPLP0, TPI1, ALDOC, ANXA4, PGAM1, and WARS) were searched for, and among available antibodies, those against PSME1, WARS, and PGAM1 showed good performance, and thus they were chosen for quantification by Western blot analysis. The levels of PGAM1, PSME1, and WARS were quantitatively investigated by Western blot analysis using NM23A as a standard (Fig. 2). Expression of PGAM1 was recognized by the presence of a single band at around 28 kDa and its protein expression was increased by 197% in bipolar disorder compared with controls (*p*<0.05). However, the levels of the other proteins were similar between bipolar disorder and controls in this case–control sample set (Fig. 2).

**Figure 3:**
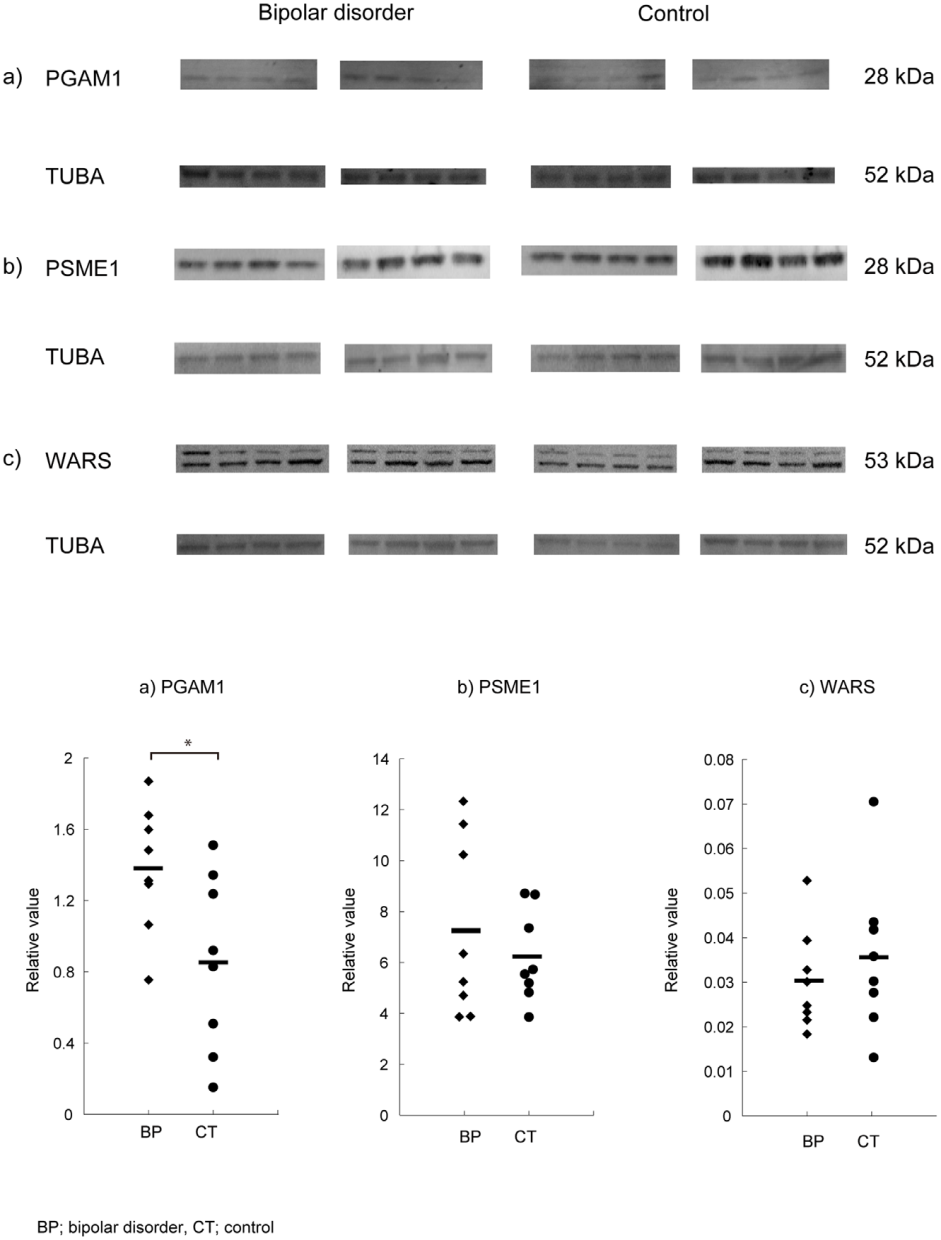
Verification of the alternation of PGAM1 using the other internal protein, tubulin alpha (TUBA). The methods are similar to the Figure 2, except that tubulin alpha (TUBA) was used for the internal standard. a) PGAM1, b) PSME1, c) WARS. Scatter plots show the ratio of each protein to an internal standard protein, TUBA, measured by densitometric scanning of the band intensities. The PGAM1/TUBA ratio was significantly higher in patients with bipolar disorder compared with controls (p<0.05). Number of the subjects is 8 for bipolar disorder and 8 for controls, respectively.

The absolute band intensity for the PGAM1 was also significantly higher in patients with bipolar disorder (0.93±0.23 [mean ± standard deviation] [arbitrary unit]) than control subjects (0.39±0.18, p<0.0005). In addition, we also performed an independent experiment using the other, more popular house-keeping protein, tubulin alpha (TUBA), as an internal standard. This analysis also showed higher PGAM1 levels in patients with bipolar disorder than controls (p<0.05) (Figure 3).

## Discussion

In this study, we identified 53 proteins that were differentially expressed between a pair of monozygotic twins discordant for bipolar disorder; 34 were up-regulated and 19 were down-regulated. The differentially expressed proteins included those previously implicated in psychiatric disorders, such as ALDOC, ENO1, and PRDX2 [10], [33]. Differences for ALDOC, ANXA4, PGAM1, PSME1, RPLP0, TPI1, and WARS between twins were regarded as robust because they were identified in three of four experiments with high scores.

To evaluate whether identified proteins might be biomarkers for bipolar disorder, we performed a case–control study for several proteins by Western blot analysis using available antibodies. An increased level of PGAM1 was observed in samples from patients with bipolar disorder. PGAM1 is an enzyme of the glycolytic pathway that catalyzes the conversion of 3-phosphoglycerate to 2-phosphoglycerate [34]. This enzyme also promotes glycolysis and ATP production via the TCA cycle and the electron transport system. Although previous studies using postmortem brains of patients with bipolar disorder and schizophrenia suggested altered protein expression of glycolysis enzymes, including PGAM1 [10], [35], the results were controversial. The differentially expressed proteins between bipolar disorder and healthy control including PGAM1, might be a clue to understand the biological basis of bipolar disorder.

To examine whether the 53 identified proteins were related to each other and constituted a global molecular network, pathway, or category, we applied IPA to our data. The results showed that the networks having a high score belonged to cell death, energy production, and glucose metabolism categories. The cell death category included the following proteins: NPM1, P4HB, LGALS3, CASP3, PDIA3, ATP5A1, GAPDH, ANXA4, HSPA5, RPLP0, UCHL1, STMN1, ENO1, ANXA5, MZB1, PSMB1, ALDOA, VDAC1, LDHA, HSPB1, and PRDX2 (Fig. 1). These results are consistent with previous studies. Benes *et al*. [36] showed increased expression of pro-apoptotic gene transcripts in postmortem brains of bipolar disorder patients. Furthermore, Herbeth *et al*. [21] indicated altered cell death and inflammation-related proteins in peripheral blood mononuclear cells and serum from patients with euthymic bipolar disorder. Brain imaging studies demonstrated reductions in the mean gray matter volume of brains from patients with bipolar disorder [37]. Previous studies reported a decreased density of nonpyramidal neurons in layer II of the anterior cingulate and a lower number of glial cells in layer III with bipolar disorder [38]. Meta-analyses of volumetric magnetic resonance imaging studies showed reduced volume of gray matter in the anterior cingulate and bilateral insula [39], [40]. Neuropathological studies of bipolar disorder showed decreases of each brain field and neuronal cells. Because mood stabilizers and antidepressants, which are used for treatment of bipolar disorder, have neuroprotective actions [5], [41], [42], it has been suggested that cells derived from patients with bipolar disorder are more vulnerable to factors related to cell death than those from controls. Patients with unipolar or bipolar depression exhibit decreased brain-derived neurotrophic factor levels [43]. Moreover, mood stabilizers have neuroprotective effects by increasing bcl-2 levels [42], [44], [45]. These findings suggest cellular vulnerability has a role in the pathology of bipolar disorder. Dysregulation of the apoptotic process found in the monozygotic twins discordant for bipolar disorder might be relevant to this hypothesis.

We examined the relationship of the identified proteins with canonical pathways and found that the proteins were related to the glycolysis pathway. The proteins included PKM2, ALDH2, ENO1, PGAM1, GAPDH, ALDOA, LDHA, and ALDOC. Glycolysis, or anaerobic respiration, is a fundamental metabolic process that produces energy for all cells. In order to maintain its functions, the brain needs an enormous amount of energy compared with other tissues. ALDOC is a brain-specific glycolysis enzyme that catalyzes the reversible aldol cleavage of fructose-1,6-biphosphate and fructose-1-phosphate to dihydroxyacetone phosphate and either glyceraldehyde-3-phosphate or glyceraldehyde [46]. In the present study, we found a decrease of the ALDOC protein level in the affected twin. However, previous reports showed that protein expression level of ALDOC was increased in the frontal cortex of patients, including those with mood disorder [35], [47]. This discrepancy might reflect differences between tissues. Moreover, we found differential expression of many essential enzymes of glycolysis such as TPI1, ALDOA, and PGAM1. A previous report using positron emission tomography showed that familial bipolar depressive patients had decreased blood flow in the cerebrum and a decreased rate of glucose metabolism in the ventral anterior cingulate cortex [37]. As indicated by an alteration in energy metabolism, compromised metabolic function has been reported in bipolar disorder [48], [49]. In these studies, alteration of mitochondrial proteins was reported. Mitochondria are involved in processes including the TCA cycle, glycolysis and gluconeogenesis, lipogenesis, and malate-asparate shuttle [50]. Thus, changes in these proteins may lead to major alterations in the energy pathways, thus affecting ATP production. Recently, many reports have suggested that mitochondrial dysfunction is involved in bipolar disorder and other psychiatric disorders [51], [52], [53]. Mitochondria are also involved in other essential processes such as apoptosis, oxidative stress, and calcium regulation [50]. Thus, a decrease in energy production due to mitochondrial dysfunction in the brains of patients with bipolar disorder may be compensated for by an increase in energy production by glycolysis. It is possible that mitochondrial dysfunction affects neuronal cell death. Further study is needed to know whether these alterations in glycolysis-related proteins are a cause or consequence of the disease process.

This is the first study to our knowledge to apply proteomics for the analysis of monozygotic twins discordant for bipolar disorder, and it has major limitations. First of all, we analyzed only a single pair of monozygotic twins. Thus, results cannot be applied to bipolar disorder in general. Another limitation is the tissue examined; that is, lymphoblastoid cells. Although brain samples may be optimal to identify molecules directly related to bipolar disorder, brain samples of twins are difficult to access. In addition, accessible tissues such as body fluid and peripheral cells such as serum, plasma, cerebrospinal fluids, saliva, urine, and peripheral blood cells should be used for biomarkers. In this study, we used lymphoblastoid cells and avoided a possible effect of medication by culturing the cells in drug-free media. However, a possibility that the effect of medication at the collection of blood last even after culturing the cells in drug-free media for a month cannot be totally ruled out. The other major limitation is the small number of case–control samples.

In summary, we performed a proteomic analysis of lymphoblastoid cells in a pair of monozygotic twins discordant for bipolar disorder. The identified proteins were mainly categorized as those involved in cell death and glycolysis. In a case–control study, protein expression of PGAM1, which is related to glycolysis, was significantly higher in patients than in healthy controls. The present findings suggest future new targets that may be relevant to the pathology of bipolar disorder. The present results need to be tested in a larger, independent sample set to reach a valid conclusion.
